# A New Theory of Menstruation

**Published:** 1891-08

**Authors:** 


A NEW THEORY OF MENSTRUATION.—Robinson (*N. Y. Medical Journal*, Jan. 24, 1891) advances the view that menstruation is governed by the ganglia situated in the uterine walls and along the tubes and closely connected with the ovaries, and that tubal motion constitutes the cycle of the menstrual period due to ganglia.

During menstruation the tube has a very marked cycle of movement. Its activity is greatest at the time of menstruation and its walls are dark-blue and crimson with injected blood. When the Graafian vesicle is ready to burst, the fimbriae are enormously enlarged. As the ovary shrinks, so the tube shrinks to its normal size to begin a new cycle of motion. The marked organ in the whole process is the tube. Menstruation might be called the motion of the tubes. The tubes do not respond to every ripening crop of eggs in the ovary alike. The ovum has degenerated, and the tube will not go through its rhythmical cycle. The theory that the lining membrane of the uterus is not a membrane, but a glandular tissue, is upheld. This "gland" plays a certain part in menstruation, but is subordinate to the action of the tubes.

It is simply the old automaton—a plexus of nerves studded over with numerous ganglia—that induces the uterus and tubes to perform their rhythmic cycle. This theory is perfectly compatible with the known fact that removal of tubes does not always prevent menstruation.—*University Med. News.*

